# Investigation of the Ototoxic Effect of Pembrolizumab Using a Rat Model

**DOI:** 10.7759/cureus.6057

**Published:** 2019-11-02

**Authors:** İhsan Kuzucu, Deniz Baklacı, İsmail Guler, Esra Özhamam Uçaryılmaz, Rauf Oğuzhan Kum, Müge Özcan

**Affiliations:** 1 Otolaryngology, Aksaray University, Faculty of Medicine, Aksaray, TUR; 2 Otorhinolaryngology, Kahramankazan State Hospital, Ankara, TUR; 3 Otolaryngology, Medipol University School of Medicine, Ankara, TUR; 4 Pathology, Ministry of Health Ankara City Hospital, Department of Pathology, Ankara, TUR; 5 Otolaryngology, Ministry of Health Ankara City Hospital, Department of Otolatyngology, Ankara, TUR

**Keywords:** programmed cell death protein-1, pembrolizumab, ototoxicity, rat

## Abstract

Objective

Programmed cell death protein-1 (PD-1) inhibitors that have been recently introduced for the systemic treatment of head and neck cancers offer the advantage of fewer side effects and more effective treatment than chemotherapy drugs. A review of the literature shows that the ototoxic side effects of the PD-1 inhibitor have not yet been fully elucidated. In this study, we aimed to investigate whether the PD-1 inhibitor has ototoxic activity using both electrophysiological and histopathological methods.

Methods

A total of 24 rats, 12 for the study group, and 12 for the control group were included in the study. The study group was administered the PD-1 inhibitor. The auditory brainstem responses (ABR) of the study and control groups were evaluated. At the end of the study, all animals were sacrificed, and their cochleae were examined by immunohistochemical staining.

Results

In the study group, the ABR values ​​were 13.95 ± 2.70 before treatment, 15.83 ± 1.94 at week 4 of treatment (p=0.024), and 15.00 ± 1.06 at week 7 (p=0.157). Furthermore, according to immunohistochemical staining, the cochlear hair cells were reduced in the study group compared to the control group.

Conclusion

It was determined that the PD-1 inhibitor showed ototoxic activity during the course of treatment, but this was spontaneously resolved during follow-up. The clinical significance of these findings should be supported by human studies.

## Introduction

Squamous cell carcinoma of the head and neck (SCCHN) is a malignancy that causes more than 300,000 deaths worldwide every year. SCCHN treatment usually requires a multidisciplinary approach involving surgery, radiotherapy, and systemic medical therapy [1].

Advancements in systemic treatment modalities have led to the development of programmed cell death protein-1 (PD-1) inhibitors that have the advantages of being more effective in appropriate indications and offering a more comfortable treatment with fewer side effects compared to chemotherapy drugs [1-3]. Pembrolizumab is a monoclonal antibody that is present in T and pro-B cells and targets PD-1. Clinically, PD-1 blockade elicits strong antitumor immune responses. Antibodies that block PD-1 ligation, such as pembrolizumab, have recently been introduced in the treatment of SCCHN, non-small cell lung cancer, kidney cell cancer, and melanoma [4]. In patients with SCCHN, the side effects of pembrolizumab therapy have been reported as fatigue, pruritus, rash, diarrhea, elevated liver function, hyponatremia, heart failure, hypothyroidism, adrenal insufficiency, and myositis [5].

Our review of the literature showed that the ototoxic side effects of pembrolizumab are not yet clearly known [6, 7]. Therefore, in this study, we investigated whether pembrolizumab has ototoxic activity based on both electrophysiological auditory brainstem responses (ABR) and histopathological data. In addition, using histopathological findings, we aimed to find an answer to the question of which part of the inner ear this possible ototoxic activity involves.

## Materials and methods

The animal experiments were commenced after the approval of the local ethics committee (protocol number 0046). Twenty-four 10-12-week-old brown rats (Rattus norvegicus) weighing 250-400 g were included in the study. The study and control groups each consisted of 12 animals, six males, and six females. Microscopic examination was performed on all subjects on day 0 (before treatment) under general anesthesia (Zeiss, OPMI 9, Germany), and rats which after examination showed natural ears were included in the study while those with tympanic membrane rupture and external auditory canal pathology were excluded. If present, the earwax plug of the rats in the outer ear was cleaned. Then, the ABR test (Eclipse 25 ABR System, Interacoustic, Denmark) was performed on the right and left ears of all rats before treatment, and the results were recorded.

Electrodes were placed as follows: ground line on the lower part of the forehead, positive line on the upper part of the forehead, one negative electrode to left ear mastoid and the other negative electrode to the right ear mastoid. In the course of the test, attention was paid to keep cables away from the recording device to the extent reasonably practice, in a separated manner and effort was made to maintain electrode-skin impedances below 5kΩ. All the animals included in the study were found to have normal ABR values ​​before the treatment. 

No treatment was applied to the control group. The study group was administered 2 mg/kg intravenous PD-1 inhibitor (BioXCell, West Lebanon, New Hampshire) through the tail of the animals twice at three-week intervals [8]. The study group received PD-1 on day 0 and at week 3, and one week after each treatment session, the ABR test was performed on both ears of the animals. After the last PD-1 injection, the animals were followed up for one month, and the final ABR test was conducted at week 7. All animals were subjected to the ABR test four times in total: day 0 (before the treatment for the study group), week 1, week 4, and week 7. At the seventh week of the study, when the last ABR was undertaken, all animals were sacrificed, and the right and left inner ear auditory region (cochlear hair cells) of each rat was histopathologically evaluated.

Tissue preparation

The cochlea of ​​each rat was kept in the fixator at 4° C for 24 hours in a 10% formaldehyde solution. The cochlea was then calcined with 10% ethylenediaminetetraacetic acid disodium salt dihydrate (pH 7.0; Muto Pure Chemicals, Tokyo, Japan) for two days at room temperature. The cochlea was dehydrated with a graduated ethanol sequence and xylene, embedded in paraffin and then cut at 3.0 µm in a midmodular plane [9, 10]. Slides containing the cochlea segments were stained with hematoxylin-eosin (H-E) (hematoxylin from Muto Pure Chemicals; eosin from Wako Pure Chemicals Industries, Osaka, Japan) to examine the structure. The samples were divided into three half-turns (basal, upper basal, apical) with a laboratory microscope (BX51; Olympus, Tokyo, Japan). Cochlear samples were photographed with a BZ-8100 light microscope. The specimens were examined by a single pathologist who was blind to the study and control groups. In histopathological evaluation, the loss of outer hair cell was evaluated as follows; 0, no degeneration (0% to 25% loss of external hair cells); +, slight degeneration (26% - 50% loss of external hair cells); ++, moderate degeneration (51% to 75% hair cell loss); and +++, severe degeneration (76% to 100% hair cell loss) [11].

Statistical analysis

Data were statistically analyzed using SPSS 21 (SPSS, Inc., IBM Company, Chicago, IL, USA). The results are presented as descriptive statistics (mean ± standard deviation [SD], median ± SD, minimum, and maximum). The normal distribution of data from each group was confirmed using the Kolmogorov-Smirnov normality test. Kruskal-Wallis test was employed to compare the groups for quantitative data. Mann-Whitney U test was used to detect the group causing the statistical difference and compare the data that did not show a normal distribution. The Friedman test was employed for intergroup comparisons of ABR data, while the Wilcoxon signed-ranks test was used for binary evaluation. P < 0.05 was considered statistically significant.

## Results

All the animals in the study and control groups subjected to ABR before treatment were observed to survive after treatment. The difference in weight loss between the study and control group animals was not statistically significant.

Ototoxicity was assessed by functional ABR measurements and histologically based on hair cell count. The pre-treatment ABR values ​​were 13.95 ± 2.70 for the study group and 13.75 ± 2.91 for the control group (p = 0.755). At week 4, the ABR values were determined as 15.83 ± 1.94 and 13.75 ± 1.30 for the study and control groups, respectively, indicating a statistically significant difference (p = 0.014). At week 7, the ABR was found to be 15.00 ± 1.06 for the study group and 13.95 ± 1.67 for the control group (p = 0.630) (Table 1).

**Table 1:** ABR values of the study and control groups throughout the treatment period ABR - auditory brainstem responses; Avg - average; SD - Standard deviation p*, Mann-Whitney U Test

ABR values		Study group (n=12)	Control group (n=12)	p*
Pretreatment (day 0)	Min - Max (Median)	10 - 20 (15)	10 - 20 (12.5)	0.755
	Avg ± SD	13.95 ± 2.70	13.75 ± 2.91	
Week 1	Min - Max (Median)	12.5 - 17.50 (15)	10 - 15.0 (12.5)	0.128
	Avg ± SD	14.58 ± 1.79	13.12 ± 1.88	
Week 4	Min - Max (Median)	12.5 - 20.0 (15)	12.5 - 15.0 (13.75)	0.014
	Avg ± SD	15.83 ± 1.94	13.75 ± 1.30	
Week 7	Min - Max (Median)	10.0 - 20.0 (15)	12.5 - 17.5 (13.75)	0.630
	Avg ± SD	15.00 ± 1.06	13.95 ± 1.67	

In the study group, the pre-treatment and week 4 ABR values of the study group did not statistically differ (p = 0.024). In the same group, the hearing loss did not significantly differ after treatment (at week 7) compared to day 0 based on ABR values (p = 0.157) (Table 2).

**Table 2:** Comparison of the ABR values of the study and control groups throughout the treatment period ABR - auditory brainstem responses; SD - standard deviation p*, Friedman test; p**, Wilcoxon signed-ranks test

ABR values		Study groups (n=12)		Control groups (n=12)
^a ^Day 0	Min - Max (Median)	10 - 20 (15)		10 - 20 (12.5)
	Mean ± SD	13.95 ± 2.70		13.75 ± 2.91
^b ^Week 1	Min - Max (Median)	12.5 - 17.50 (15)		10 - 15.0 (12.5)
	Mean ± SD	14.58 ± 1.79		13.12 ± 1.88
^c ^Week 4	Min - Max (Median)	12.5 - 20.0 (15)		12.5 - 15.0 (13.75)
	Mean ± SD	15.83 ± 1.94		13.75 ± 1.30
^d ^Week 7	Min - Max (Median)	10.0 - 20.0 (15)		12.5 - 17.5 (13.75)
	Mean ± SD	15.00 ± 1.06		13.95 ± 1.67
	p*	0.008		0.630
	p** Weeks in the study group ^a-b^p = 0.180 ^a-c^p = 0.024 ^a-d^p = 0.157		^b-c^p = 0.014 ^b-d^p = 0.655 ^c-d^p = 0.038	

**Figure 1:**
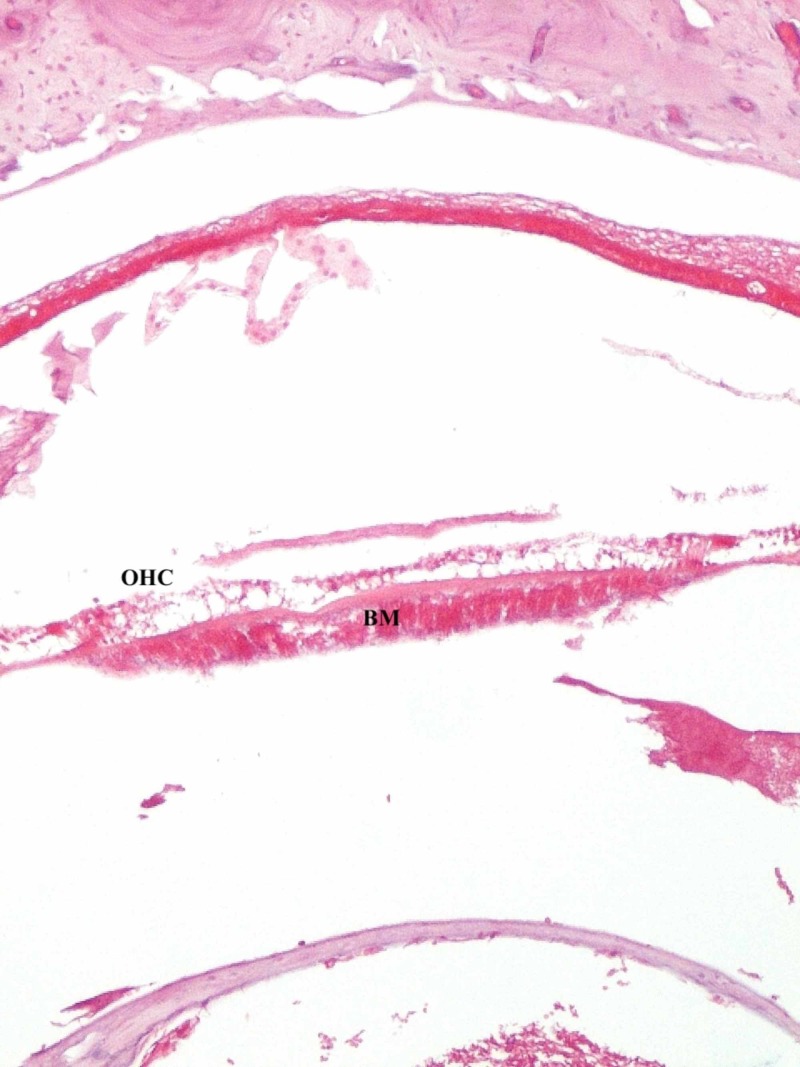
Slight loss of outer hair cell OHC - outher hair cell; BM - basilar membrane

**Figure 2:**
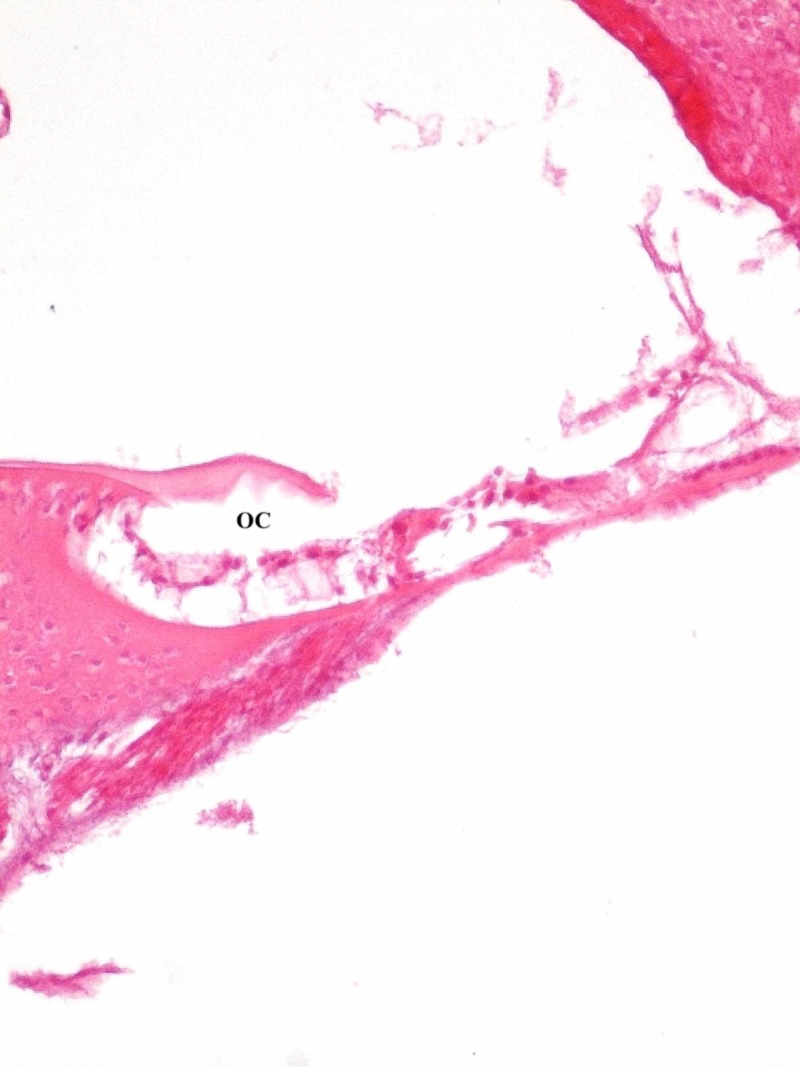
Flattened organ of Corti OC - organ of Corti

Light microscopy showed that outer hair cells were mostly protected in control groups. There was a slight loss of outer hair cell (Figure 1) and flattening of the organ of Corti (Figure 2) in the study group.

## Discussion

This study investigated the ototoxic effects of PD-1 inhibitor using histopathological and electrophysiological methods. According to the results of the study, PD-1 inhibitor caused hearing loss at the second dose of treatment; however, this was observed to be spontaneously resolved in the follow-up sessions. At the same time, the histopathological examination revealed a decrease in hair cells.

In recent years, immunotherapy drugs have been used for the treatment of many cancers. Cancer immunotherapy involves various therapeutic approaches that aim to boost the immune system and destroy tumor cells [12]. However, since cancers are derived from the patient’s own cells, tumor cells retain many natural autoimmune defense mechanisms that can inhibit tumor immune destruction. Disruption of these autoimmune defense mechanisms has been shown to be highly successful in eliciting antitumor responses to many types of cancer. One of the well-known immune checkpoint pathways is PD-1 [13]. Pembrolizumab, an approved IgG 4 monoclonal antibody for the treatment of advanced-stage melanoma and non-small cell lung cancer, also offers promising results in other malignancies [14]. However, excessive accumulation of PD-1 antibodies in the liver and kidneys has limited the use of these agents in certain cases [15]. Today, researchers are still seeking a solution to this problem, and the use of PD-1 inhibitor drugs in other cancers is increasing.

In the related literature studies, the effective dose of pembrolizumab was reported and accepted as 2 mg/kg. When pembrolizumab was administered at a dose of more than 2 mg/kg, there was only a minimal increase in drug activity [8]. The half-life of pembrolizumab is 22 days; therefore, it is recommended that the drug be given in three-week periods [5]. In the current study, we used pembrolizumab at the recommended dose of 2 mg/kg at three-week intervals to investigate the presence of ototoxic activity of pembrolizumab in clinical practice. When we review the literature, it has been reported that sensorineural hearing loss due to pembrolizumab use is usually bilateral, moderate-severe and hearing loss develops within the first three weeks after initiation of treatment [16, 17]. Therefore, the duration of our study was four weeks.

In a study by Spielbauer et al. using a murine model, the cisplatin-treated group was compared with the group that was administered cisplatin plus PD-1 inhibitor [18]. Hearing loss was reported to be higher in the cisplatin plus PD-1 inhibitor group, but the difference between the two groups was not statistically significant. In the current study, although there was no significant difference in ABR between the study and control groups, we found that the hearing ability decreased in the study group. Furthermore, histopathologically, we found a decrease in hair cells in the inner ears of the rats in the study group compared to the control group. The data we obtained are consistent with those reported in the literature. We recommended that this study is repeated in human beings over a longer follow-up period.

It is also important to enumerate some of the limitations of this study. For example, the number of animals was limited. Furthermore, light microscopy is known to have limitations even when the best material is used and electron microscopy is essential to see the inner ear structures with much better resolution. Thus, further investigations using transmission and scanning electron microscopy methods are needed. 

## Conclusions

According to the data obtained from the rat model, pembrolizumab showed signs of ototoxicity during the course of the treatment, but hearing loss was spontaneously resolved during follow-up. The clinical significance of these findings should be confirmed by human studies. It is expected that in the future, otolaryngologists will see a large increase in the number of patients treated with chemoimmunotherapy. These patients should be monitored for hearing loss and other toxicities.
